# Metabolite Profiling of the Microalgal Diatom *Chaetoceros Calcitrans* and Correlation with Antioxidant and Nitric Oxide Inhibitory Activities via ^1^H NMR-Based Metabolomics

**DOI:** 10.3390/md16050154

**Published:** 2018-05-07

**Authors:** Awanis Azizan, Muhammad Safwan Ahamad Bustamam, M. Maulidiani, Khozirah Shaari, Intan Safinar Ismail, Norio Nagao, Faridah Abas

**Affiliations:** 1 Laboratory of Natural Products, Institute of Bioscience, Universiti Putra Malaysia, 43400 Serdang, Selangor, Malaysia; awanis_azizan@yahoo.com (A.A.); safwan.upm@gmail.com (M.S.A.B.); maulidiani@upm.edu.my (M.M.); khozirah@upm.edu.my (K.S.); safinar@upm.edu.my (I.S.I.); 2 Department of Chemistry, Faculty of Science, Universiti Putra Malaysia, 43400 Serdang, Selangor, Malaysia; 3 Laboratory of Marine Biotechnology, Institute of Bioscience, Universiti Putra Malaysia, 43400 Serdang, Selangor, Malaysia; norio.nagao@upm.edu.my; 4 Department of Food Science, Faculty of Food Science and Technology, Universiti Putra Malaysia, 43400 Serdang, Selangor, Malaysia

**Keywords:** *Chaetoceros calcitrans*, metabolite profiling, ^1^H nuclear magnetic resonance (NMR) spectroscopy, antioxidant, nitric oxide inhibitory

## Abstract

Microalgae are promising candidate resources from marine ecology for health-improving effects. Metabolite profiling of the microalgal diatom, *Chaetoceros calcitrans* was conducted by using robust metabolomics tools, namely ^1^H nuclear magnetic resonance (NMR) spectroscopy coupled with multivariate data analysis (MVDA). The unsupervised data analysis, using principal component analysis (PCA), resolved the five types of extracts made by solvents ranging from polar to non-polar into five different clusters. Collectively, with various extraction solvents, 11 amino acids, cholesterol, 6 fatty acids, 2 sugars, 1 osmolyte, 6 carotenoids and 2 chlorophyll pigments were identified. The fatty acids and both carotenoid pigments as well as chlorophyll, were observed in the extracts made from medium polar (acetone, chloroform) and non-polar (hexane) solvents. It is suggested that the compounds were the characteristic markers that influenced the separation between the clusters. Based on partial least square (PLS) analysis, fucoxanthin, astaxanthin, violaxanthin, zeaxanthin, canthaxanthin, and lutein displayed strong correlation to 2,2-diphenyl-1-picrylhydrazyl (DPPH) free radical scavenging and nitric oxide (NO) inhibitory activity. This metabolomics study showed that solvent extractions are one of the main bottlenecks for the maximum recovery of bioactive microalgal compounds and could be a better source of natural antioxidants due to a high value of metabolites.

## 1. Introduction

Microalgae are microscopic organisms inhabiting almost every marine environment, including freshwater ecosystems, such as ponds and lakes, and saltwater ecosystems, such as the oceans around the world and even the Antarctic [1,2]. Numerous studies showed that microalgae contain high level of carotenoids [3], chlorophyll [4], phenolic content and polyunsaturated fatty acids (PUFAs) [5,6]. Carotenoids and chlorophyll are well recognized as natural antioxidant pigments due to their capability to scavenge free radicals, reduce oxidative stress and enhance immunization [7]. PUFAs might be used for preventing cardiovascular disease [8]. Additionally, the fossilized remains of the microalgal diatom known as diatomite is also commercially valuable as a filter, absorbent, anti-caking agent, and material for insulation [9]. Thus, microalgae exhibit high potential as sources of biological activities in the food, pharmaceutical, environmental and cosmetic industries.

*Chaetoceros calcitrans*, the species belonging to the family *Bacillariophyceae,* was used in this study, since it contains high amounts of natural antioxidant pigments (carotenoids and chlorophylls) and PUFAs [10,11]. Recently, Foo and co-workers [11] identified fucoxanthin from this diatom extract as being influential for antioxidant activity. The group also suggested that the outstanding antioxidative activity of fucoxanthin isolated from the dichloromethane fraction of *C. calcitrans* may be attributed to the presence of allenic bonds at C-7′ position, conjugated carbonyl, 5,6-monoepoxide and the acetyl groups, in which the excited electrons in radicals are transferred [12]. Another group, Nigjeh et al. [13], discovered that exposing crude ethanol extracts of this indigenous microalgae, *C. calcitrans* on human breast cancer cell lines, MCF-7, resulted in apoptosis and decreased proliferation of these cells. Goh et al. [14] also reported that through an apoptosis determination test the ethyl acetate (CEA) extract of *C. calcitrans* showed remarkable cytotoxicity effect on parental cancer cell lines, MDA-MB-231 cells. Other closely related species, *Chaetoceros muelleri* and *Chaetoceros lauderi* displayed antimicrobial and antibacterial effects due to elevated amounts of eicosapentaenoic acids (EPA) [15,16]. Nevertheless, there is no available information regarding the anti-inflammatory activity of *C. calcitrans*.

Aside from potential therapeutic applications for *C. calcitrans*, it has also been reported that this microalga is suitable as a valuable feed supplement or substitute for conventional animal feed sources. Microalgal biotechnology has made it possible to produce this microalga commercially via continuous production even though it is not very easy to grow on a large scale [17]. Metabolite extraction is a key stage in the workflow of a metabolomics study. In this regard, the choice of solvents used for extraction is critical since the main aim of this step is to extract as wide a spectrum of chemical structures as possible from the metabolites [18]. Hence, solvents with polarity indexes closest to the desired polarities of the metabolites are usually selected.

To evaluate the contribution of metabolites in microalgae to their antioxidant activity, a mostly targeted approach was used [19,20]. In the targeted approach, only the desired metabolites were analyzed and, generally, the number of metabolites is low. However, the extraction of microalgae can contain a lot of metabolites and some of the metabolites may contribute to their activity. Thus, comprehensive and holistic analysis (the un-targeted approach) is necessary. ^1^H nuclear magnetic resonance (NMR)-based metabolomics have been used widely for un-targeted analysis to evaluate the correlation of the identified metabolites in ^1^H NMR spectra of plant extracts to their biological activities, such as antioxidant [21], anti-inflammation [22], and antidiabetic [23] activities. However, there is no data on the metabolic discrimination of *C. calcitrans* extracted by different solvents and the biological activity correlation using the metabolomics approach. Therefore, this study aims to establish metabolite profiles in the microalgal diatom *C. calcitrans* extracted by different polarity solvents methanol (Me), 70% ethanol (70% Et), acetone (Ac), chloroform (Ch) and hexane (He), and to correlate these profiles to biological activities using ^1^H NMR-based metabolomics. In addition, relative quantification and possible metabolite biosynthesis pathways are also suggested.

## 2. Results and Discussion

### 2.1. Assignments of Metabolites by 1D Nuclear Magnetic Resonance (NMR) and 2D NMR Spectra in Microalgal Crude Extracts

**Figure 1:**
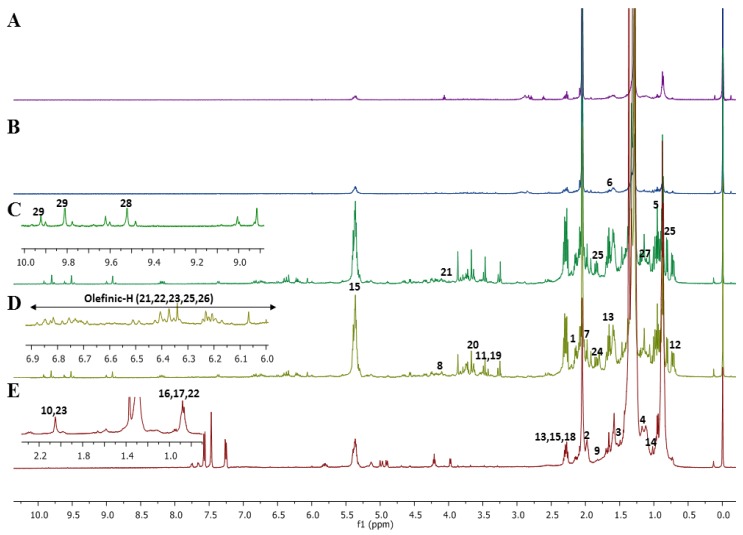
Representative 500 MHz ^1^H nuclear magnetic resonance (NMR) spectra of *Chaetoceros calcitrans* in different solvents extraction: (**A**) 70% ethanol; (**B**) methanol; (**C**) acetone; (**D**) chloroform; (**E**) hexane. Identified metabolite signals: 1, glutamate; 2, proline; 3, alanine; 4, valine; 5, isoleucine; 6, leucine; 7, methionine; 8, choline; 9, lysine; 10, glutamine; 11, glycine; 12, cholesterol; 13, palmitic acid; 14, stearic acid; 15, oleic acid; 16, linolenic acid; 17, α-linolenic acid; 18, arachidic acid; 19, glucose; 20, sucrose; 21, myo-inositol; 22, fucoxanthin; 23, astaxanthin; 24, canthaxanthin; 25, lutein; 26, zeaxanthin; 27, violaxanthin; 28, chlorophyll *c*_1_; 29, chlorophyll *a*.

Previous studies on marine microalgae have reported an abundance of primary metabolites, including lipids, fatty acids, amino acids and simple sugars. However, there is limited data on secondary metabolites in microalgae. Assignment of peak signals in the ^1^H NMR and 2D NMR spectra were based on previously reported literature [24,25,26,27,28] and by comparison with standard online databases (freely available), such as the Human Metabolome Database (HMDB) at http://www.hmdb.ca/, the Biological Magnetic Resonance (BMR) database at http://www.bmrb.wisc.edu/ and PubChem at https://pubchem.ncbi.nlm.nih.gov/. Figure 1 shows the representative ^1^H NMR spectra of the different solvent extracts of *C. calcitrans*. The spectra showed the presence of several different classes of metabolites, including carotenoids, fatty acids, amino acids, organic acids, and sugars. The peak chemical shifts for the corresponding 29 identified metabolites are summarized in Table 1.

Notable variations among the different solvent extracts were observed for the regions 6.0–7.0 ppm, 7.0–8.0 ppm, and 8.2–10.0 ppm, which were the characteristic chemical shift areas for carotenoids and of chlorophyllic constituents. Differences were also detected in the high-field regions 0.5–2.5 ppm and 3.0–4.0 ppm, which corresponded to the regions for amino acids and carbohydrates [25,26]. Signals for amino acids and carbohydrates display characteristic amine protons, methyls, methine groups and anomeric protons (for carbohydrates) which were detected in most of the polar and medium-polar solvent extracts [28]. However, peak congestion in these regions makes the assignment of compounds very challenging. In the present study, 10 amino acids and two carbohydrate constituents were identified in these spectral regions. The identification was further supported by 2D experiments (*J*-resolved, heteronuclear multiple-bond correlation, (HMBC) spectroscopy).

Acetone, chloroform and hexane extracts of *C. calcitrans* were among the solvent extracts that showed signals for carotenoids, specifically the regions 6.0–7.0 ppm (olefinic protons) and 0.8–2.5 ppm (aliphatic protons) [24]. Generally, there are two types of carotenoids, which include one that forms from only carbon and hydrogen and are called carotenes, whereas another type, which contain oxygen, are called xanthophylls [29,30]. The proton resonances assignable to olefinic protons were observed at 6.75, 6.66, 6.58, 6.41, 6.13, and 6.07 ppm for fucoxanthin, 6.20–6.70 ppm for canthaxanthin and astaxanthin, and 6.67–6.57 ppm for lutein. Multiplet signals were observed between 6.10–6.40 ppm for zeaxanthin. Fucoxanthin and astaxanthin identification have been confirmed by 2D NMR. The *J*-resolved spectrum observed signals at 2.07 and 2.01 ppm corresponding to methyls proton of fucoxanthin and astaxanthin, respectively (Figure S1). From the HMBC spectrum, a 2 *J* correlation between the proton signal at 2.07 ppm (H-19) to the carbonyl signal at 200 ppm (C-8) and 3 *J* correlation between the proton at 3.78 (H-3′) ppm to methine carbon signal at 65 ppm (C-5′) in cyclohexene ring could be seen which confirmed the assignment of fucoxanthin (Figure S2A). As for astaxanthin, correlations were observed between the proton signal at 2.01 ppm (H-17) with the methyl carbon at 17 ppm (C-2) in cyclohexene ring (Figure S2A). Unfortunately, none of the 2D experiments were able to confirm precisely the resonances of the zeaxanthin, canthaxanthin, lutein and violaxanthin due to their low concentrations in the extracts.

The ^1^H NMR spectra of the hexane extract showed signals characteristic of fatty acids. The signals were close to the reported signals for PUFAs, such as arachidic acid [C20:0], α-linolenic acid [C18:3], linoleic acid [C18:2], stearic acid [C18:0], oleic acid [C18:1] and palmitic acid [C16:0] [25,26]. The signals corresponding to α-linolenic acid [C18:3], linoleic acid [C18:2], and oleic acid [C18:1] were deduced based on the presence of the olefinic protons (5.30–5.40 ppm), protons attached to the bisallylic carbons (129–132 ppm). The *J*-resolved spectrum showed a multiplet signals of the olefinic protons at 5.30–5.40 ppm and the HMBC spectrum revealed that these signals were correlated to methylene carbon signals at 25–27 ppm which signified the characteristic of PUFAs (Figures S1 and S2). The difference in double-bond positions as well as double-bond configuration (i.e., either cis or trans) were also considered in order to determine the fatty acid profile. Moreover, to identify tentatively the presence of palmitic acid, stearic acid and also arachidic acid, the signal of methylene group (CH_2_) protons, observed at 1.20–1.40 ppm, was used to discriminate the fatty acid components. The assignment of the fatty acid ^1^H NMR resonances was further supported by 2D NMR data as shown in Figures S3 and S4. The spectral features and the chemical shift assignments were compared with the previous reported literature.

Several resonances belonging to chlorophyll *c*_1_ (Chl *c*_1_) and chlorophyll *a* (Chl *a*), which are accessory pigments, were seen in many diatoms. NMR signals for these pigments can be found in the 8–10 ppm region of the ^1^H NMR spectra. An important general feature of these pigments is the presence of protons on the chlorophyll macrocycle, which can cause a “ring current effect” [31]. Signals at 9.96, 9.89, 9.81 and 8.29 ppm were assigned to chlorophyll *c*_1_ and the signal at 9.52 ppm was assigned to chlorophyll *a*. From the HMBC spectrum, 2 *J* correlation was observed between the proton signal at 9.52 ppm and carbon signal at 125 ppm in the pyrroline ring of the chlorophyll *a* (Figure S2A). As in the case of chlorophyll *c*_1_, HMBC was unable to detect the connectivity between ^1^H and ^13^C due to low concentrations.

### 2.2. Classification of Different Solvent Extracts by Principal Component Analysis (PCA)

**Figure 2:**
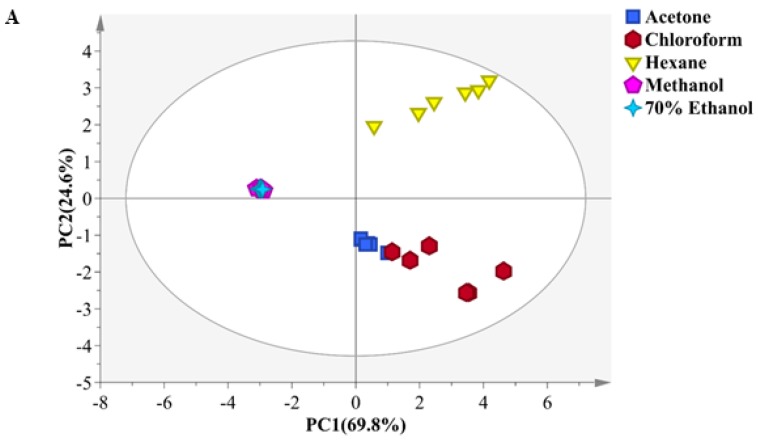

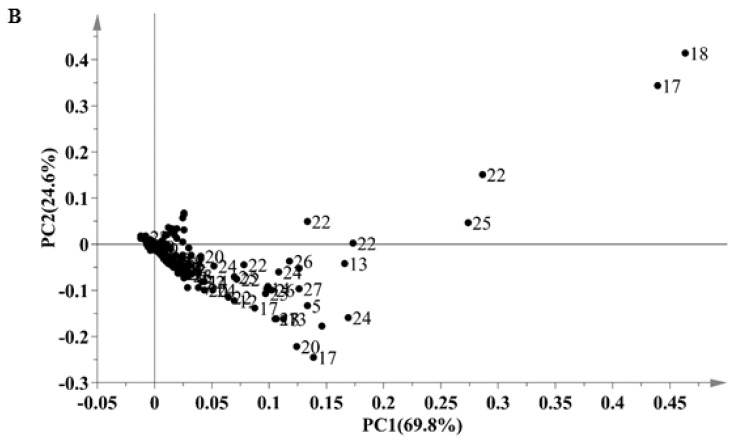
Score plot of principal component analysis (PC1 versus PC2) in (**A**) and loading plot (**B**) obtained from five different solvent extractions of *Chaetoceros calcitrans*. The plot elipse represents 95% hotelling T2 confidence.

Assignment of metabolites in the ^1^H NMR and 2D NMR spectra of acetone, chloroform, hexane, methanol and 70% ethanol extracts revealed significant variations in metabolite constituents. Consequently, multivariate data analysis was used to analyze the differences in metabolites present in the solvent extracts. It was evident from the PCA model that the 30 samples were clustered into five groups with good fitness (R2X cum) and high predictability (Q2 cum) values of 0.98 and 0.97, respectively (Figure 2A). In addition, no outliers were seen in the score plot. The first principal component (PC1) accounted for 69.8% of the variance, while the second principal component (PC2) ccounted for 24.6% of the variance, cumulatively explaining the total variance of 94.4%. Thus, the metabolite profiles of the five solvent extracts were clearly different from each other. The acetone, chloroform and hexane extracts were well separated from the rest of the extracts by first principal component (PC1), while the hexane, methanol and 70% ethanol extracts were separated from the acetone and chloroform extracts by second principal component (PC2). Interestingly, the spectral signals for methanol and 70% ethanol extracts were in the same cluster, indicating that they have the same chemical profiles.

The loading plot highlighted the potential marker compounds responsible for the grouping. The loading plot with PC1 and PC2 for the five solvent extractions is presented in Figure 2B. Further detailed examination of the loading plot showed that the acetone and chloroform extracts contained significantly higher amounts of carotenoids and chlorophyll pigments compared to the other extracts. The carotenoids detected were fucoxanthin, canthaxanthin, astaxanthin, violaxanthin, zeaxanthin and lutein, while the chlorophyll detected were Chl *a* and Chl *c*_1_. Signals for the carotenoids were resonated in the spectral region between 6.10–6.80 ppm, which were contributed by the olefinic and methyl proton. The significantly high amounts of carotenoids and chlorophyll pigments indicated their potential as antioxidants. Fatty acid, particularly α-linolenic acid, identified from the characteristic signal at 1.30 ppm, was shown to be present in relatively higher amounts in the hexane extract in comparison to the other extracts. Meanwhile, the discriminating factors for the 70% ethanol and methanol extracts from the other extracts were mainly due to amino acids. These compounds are primary metabolites in lower plants and have been reported to contribute to numerous bioactivities.

### 2.3. Relative Quantification

To further validate the results, the amount of metabolites detected from the ^1^H NMR spectra were relatively quantified based on the variable importance in the projection (VIP) values of its partial least squares discriminant analysis (PLSDA) model (Figure S3B). In this regard, metabolites with VIP value greater than 0.5 (considered as top-ranked metabolites) were selected to determine if they might influence the clustering of the X-variable (identified metabolites) in the PCA loading plot [32].

**Figure 3:**
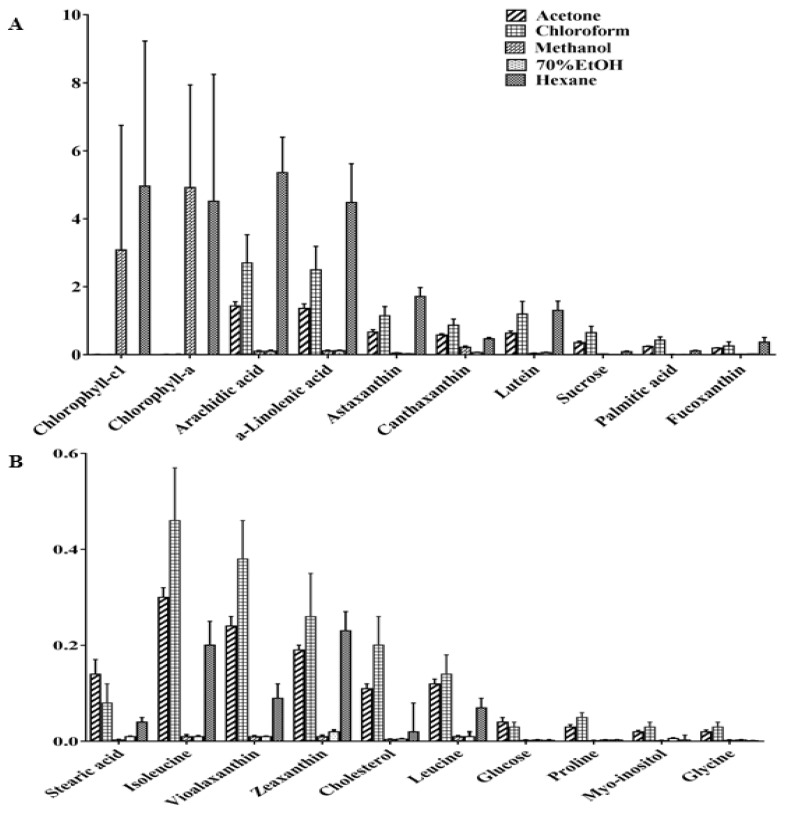
Relative quantification of the identified compounds (**A**) chlorophyll-c1, chlorophyll-a, arachidic acid, α-linolenic acid, astaxanthin, cantaxanthin, canthaxanthin, lutein, sucrose, palmitic acid and fucoxanthin, (**B**) stearic acid, isoleucine, violaxanthin, zeaxanthin, cholesterol, leucine, glucose, proline, myo-inositol and glycine of different extraction solvents from *Chaetoceros calcitrans* based on the mean peak area of ^1^H NMR signals. Chemical shifts (ppm) used for the relative quantification are chlorophyll c1 (9.82), chlorophyll a (9.5), arachidic acid (1.26), α-linolenic acid (1.3), astaxanthin (1.34), canthaxanthin (2.02), lutein (0.86), sucrose (5.38), palmitic acid (1.66), fucoxanthin (1.22), stearic acid (1.78), isoleucine (0.94), violaxanthin (0.98), zeaxanthin (1.1), cholesterol (0.7), leucine (1.7), glucose (3.5), proline (4.1), myo-inositol (3.62) and glycine (3.54). Data presented are based on the mean of six replicates each of the solvent systems (acetone, chloroform, hexane, methanol, 70% ethanol) ± standard deviation (SD).

Figure 3A,B show the graph with the metabolites and their relative quantities in the different extracts. The hexane extract was characterized by significantly higher amounts of arachidic acid (binned at 1.26 ppm), α-linolenic acid (binned at 1.30 ppm), astaxanthin (binned at 1.34 ppm), lutein (binned at 0.86 ppm), fucoxanthin (binned at 1.22 ppm), and Chl *c*_1_ (binned at 9.82 ppm) in comparison to the other extracts. In addition, the chloroform extract contained higher levels of canthaxanthin (binned at 1.22 ppm), palmitic acid (binned at 1.66 ppm), stearic acid (binned at 1.78 ppm), isoleucine (binned at 0.94 ppm), violaxanthin (binned at 0.98 ppm), zeaxanthin (binned at 1.10 ppm), cholesterol (binned 0.70 ppm) and leucine (binned at 1.70 ppm) in comparison to the acetone extract. However, lower numbers of metabolites were observed in the 70% ethanol extract. These results indicated that medium and non-polar solvents such as chloroform, acetone and hexane were the best solvents to use for extraction of the microalgae, allowing a good recovery of the hydrophobic compounds such as fatty acids, lipids and pigments. Furthermore, according to the Tukey honest significant difference (HSD) pairwise comparison (Table S1), the carotenoid contents of the chloroform, acetone and hexane extracts, represented by spectral signals of fucoxanthin, astaxanthin, violaxanthin and lutein, were highly significant (*P* ≤ 0.001) compared to those in the methanol and 70% ethanol extracts. The amounts of fatty acids, such as arachidic acid and α-linolenic acids, were significantly higher (*P* ≤ 0.001) in the hexane extract when compared to the other extracts. However, the higher amount palmitic and stearic acids were highly significant (*P* ≤ 0.001) in the acetone and chloroform extracts. The amount of the amino acid such as isoleucine was significantly higher (*P* ≤ 0.001) in all the extracts other than the 70% ethanol extract.

### 2.4. Effect of Different Solvent Extractions on Total Phenolic Content (TPC), 2,2-Diphenyl-1-Picrylhydrazyl (DPPH) Radical Scavenging and Nitric Oxide Inhibitory Activities

**Figure 4:**
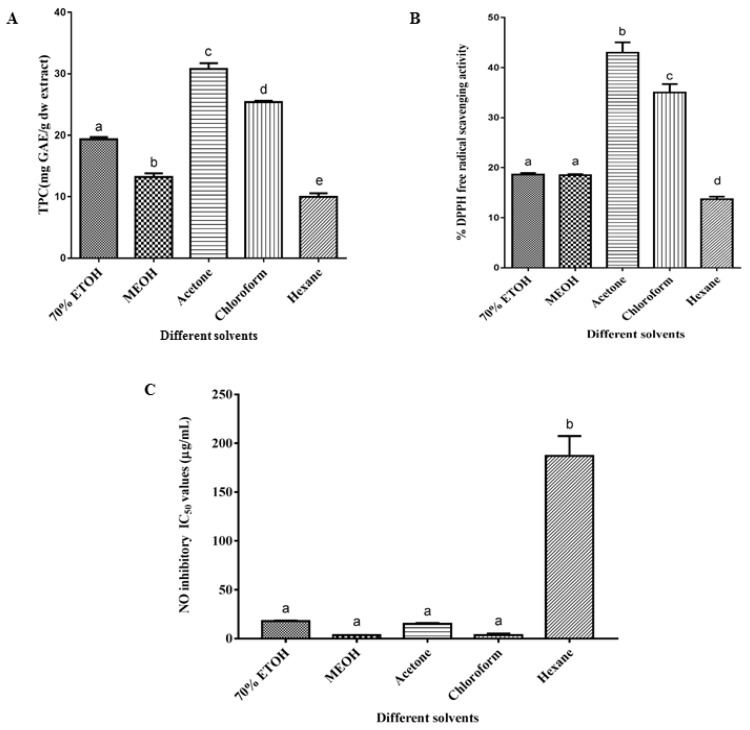
Total phenolic content (**A**), percentage of DPPH free radical scavenging activity (**B**), and NO inhibitory IC50 (**C**) of the diatom *Chaetoceros calcitrans*. Values are the mean of 6 replicates ± SD. Means with different letters indicate the significant differences (*P* < 0.05; *n* = 6) differences. IC50 Quercetin = 4.73 µg/mL. IC50 Curcumin = 6.10 µg/mL.

As shown in Figure 4, the five different solvent systems contributed variation to the total phenolic content (TPC), 2,2-diphenyl-1-picrylhydrazyl (DPPH) free radical scavenging and nitric oxide inhibitory activities of *C. calcitrans*. Acetone and chloroform extracts exhibited comparably high percentages of DPPH free radical scavenging activity (Figure 4A). The percentage inhibition for acetone extract was 43.01% whereas for chloroform extracts with 35.03% of inhibition. There were significant differences (*P* < 0.05) in all solvent systems with the exception that ethanol and methanol extracts, 70% ethanol and methanol extracts of *C. calcitrans* were statistically similar with only 18.61 and 18.51% of ability to scavenge the free radicals. On the other hand, hexane extract showed the lowest DPPH scavenging activity with only 13.71%. The results in the present study are contrary to the results reported from previous study by Foo et al. [10] where they found the methanol extracts exhibited better free radical scavenging activity in comparison to the other solvent systems. This contradictory result on scavenging activity may vary the metabolites’ yield due to different microalgae growth conditions, origin of the strains, methods during microalgal cultivation and harvesting, as well as temperature and time for extraction [20,33].

The results of DPPH radical scavenging activity showed almost the same par with TPC for the different solvent extractions (Figure 4B). It was observed that acetone possessed high total phenolic content with 30.79 mg GAE/g dw extract compared to chloroform with 25.41 mg GAE/g dw extract. When comparing 70% ethanol and methanol extracts of *C. calcitrans*, 70% ethanol contained higher TPC with a value of 19.34 mg GAE/g dw extract. Hexane extract had the lowest TPC value of 9.95 mg GAE/g dw extract compared to the other solvent extractions. Statistically, there were significant differences (*P* < 0.05) in all solvent systems. In addition to having activity for DPPH free radical scavenging and the presence of phenolic compounds, the results also demonstrated that the extracts of *C. calcitrans* showed the inhibitory effects of NO, as shown in Figure 4C. The IC50 values of some of the extracts indicated higher activity than cucurmin. However, unlike the results of TPC and DPPH scavenging activity, the chloroform, methanol, acetone and 70% ethanol extracts exhibited stronger NO inhibitory activity with IC50 values of 3.46, 3.83, 15.35, 17.94 µg/mL compared to the hexane extract with a IC50 value of 187.7 µg/mL. There was no significant difference (*P* > 0.05) between these four extracts but higher significant difference (*P* < 0.05) for hexane extracts. The inhibition was not caused by the toxicity as the values of cell viability for all extracts were more than 80%.

### 2.5. The Correlation Study between the Metabolites and Biological Activities in C. calcitrans Extracts

**Figure 5:**
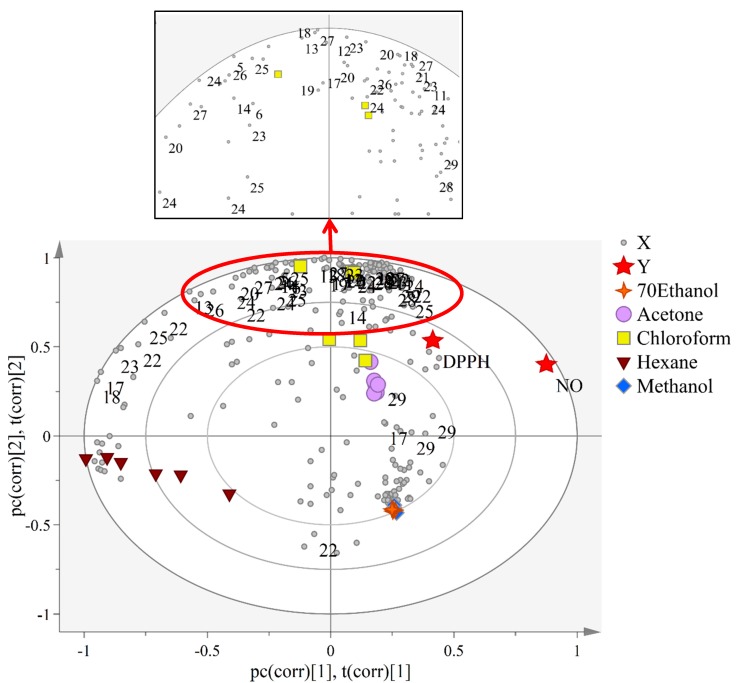
Partial least square (PLS) loading biplot of bioactivities represented by DPPH and NO inhibitory activities. Expansion of region, showing the overlapped numbers of tentatively identified compounds. The assigned compounds are in small circle shapes and grey in color. Assignments: 5, leucine; 6, isoleucine; 11, glycine; 12, cholesterol; 13, palmitic acid; 14, stearic acids; 17, α-Linolenic acid; 18, arachidic acid; 19, glucose; 20, sucrose; 21, myo-inositol; 22, fucoxanthin; 23, astaxanthin; 24, canthaxanthin; 25, lutein; 26, zeaxanthin; 27, violaxanthin 28, chlorophyll *a*; 29, chlorophyll *c*_1_.

To understand the metabolites that correlated to the studied bioactivities, partial least squares projection to latent structures (PLS), a supervised approach was used to relate data of the independent variables (*X*-matrix) to data of the dependent variables such as DPPH and NO inhibitory activities [34]. Therefore, metabolites that serve as biomarkers can be suggested through this approach. As shown in the PLS biplot (Figure 5), it can be seen that five clusters were shown without outliers. The model showed the good fitness (R2Y) value of 0.96, whereas the predictive ability (Q2) was 0.91. The *Y*-variables (DPPH and NO inhibitory activities) were clustered on the positive side of the plot, which are close to the chloroform and acetone extracts.

Carotenoids such as fucoxanthin, astaxanthin, violaxanthin, zeaxanthin, canthaxanthin and lutein that were concentrated in chloroform and acetone extracts are the compounds responsible for influencing the bioactivities of *Y*-variables of *C. calcitrans* samples. Other compounds including Chl *c*_1_, arachidic acid and α-linolenic acid are also shown to be correlated. To test the credibility of the PLS model, permutation tests with 100 permutations were done. The permutation test result of NO inhibitory (Figure S4A) showed the *Y*-intercepts of *R^2^* and *Q^2^* were 0.0839 and −0.307, whereas DPPH free radical scavenging activity (Figure S4B), the *Y*-intercepts of *R^2^* and *Q^2^* were 0.189 and −0.359, which showed that both of the constructed model were valid and not over fitting. Figure S4C,D showed the relationship between the observed versus predicted plots of the *Y*-variables, NO inhibitory and DPPH free radical scavenging activity, using a regression line with its *R^2^* value of 0.97 and 0.63, respectively. In addition, for NO inhibitory (Figure S4C) showed relatively low root mean square error of estimation **(**RMSEE) value of 3.159 and root mean square error of cross validation (RMSECV) value of 4.563 whereas for DPPH (Figure S4D) showed the RMSEE value of 6.891 and RMSECV value of 7.394, indicating the model is good and can be used for prediction.

### 2.6. Metabolite Network Analysis in Diatom C. calcitrans

In the present study, 11 amino acids, including glutamate, glutamine, proline, alanine, valine, leucine, isoleucine, lysine, methionine, glycine and choline were detected. These amino acids were manufactured from the cellular carbon (C) and nitrogen (N) metabolisms through glycolysis, gluconeogenesis, respiration and the citric acid cycle [35]. However, based on the VIP values, only isoleucine, leucine, glycine and proline were selected for relative quantification.

In diatoms, isoleucine is derived from the reduction of threonine. This amino acid production starts with synthesis of aspartate, which contained oxaloacetic acid as the backbone. Isoleucine was significantly more abundant (*P* ≤ 0.001) in all the solvent extracts, except for the methanol extract. Leucine was significantly (*P* ≤ 0.001) higher in hexane, methanol and 70% ethanol extracts compared to the acetone and chloroform extracts. Its metabolism in diatoms occurs through the synthesis of pyruvate. Glutamate has also been identified as a precursor for proline biosynthesis in diatoms [35]. Diatoms utilize nitrogen-rich amino acids for growth and cell-defense. Proline was found to be significantly higher (*P* ≤ 0.001) in acetone and chloroform extracts compared to other solvent extracts of *C. calcitrans*.

**Figure 6:**
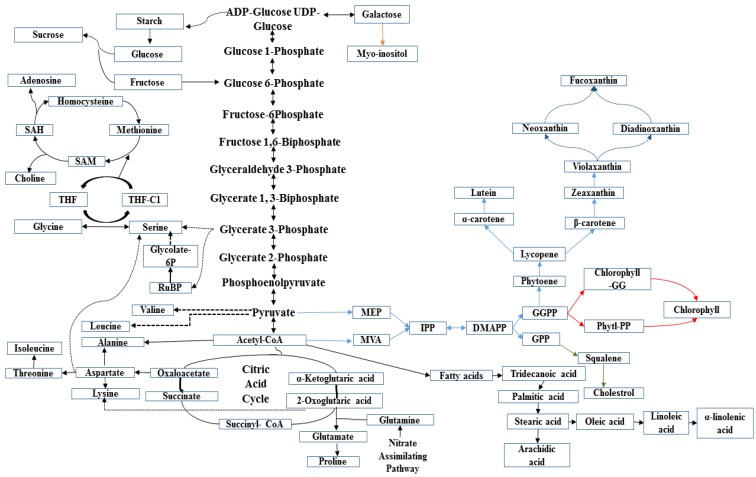
Metabolic map of different biosynthetic pathways (amino acids, carbohydrates, fatty acids, cholesterol, photosynthetic pigments) for various functions in the diatom *Chaetoceros calcitrans*. Abbreviations: THF, tetrahydrofolate; SAM, *S*-adenosyl methionine; SAH, *S*-adenosyl homocysteine; RuBP, Ribulose 1,5-bis phosphate; DMAPP, dimethylallyl diphosphate; GGPP, geranylgeranyl diphosphate; GPP, geranyl diphosphate; IPP, isopentenyl diphosphate; MEP, 2-*C*-methyl-d-erythritol 4-phosphate; MVA, mevalonate.

Many diatomic microalgae produce abundant carbohydrate and sugar alcohols. As shown in Figure 6, these metabolites stem from pathways such as glycolysis, gluconeogenesis and sugar-like metabolism. Acetone and chloroform extracts of *C. calcitrans* accumulated significantly higher (*P* ≤ 0.001) amounts of the end products of carbohydrate biosynthesis pathways, such as sucrose and glucose. These sugars are found in medium polar solvents as they might be present in the form of glycosides, which are bonded to the other compounds. A number of sugar-alcohol metabolites, in particular the end products of sugar breakdown, were also detected in marine microalgae. Cyclitols, particularly myo-inositol was found to be significantly (*P* ≤ 0.001) more abundant in acetone and chloroform extracts. This storage compound plays an important role in preserving the bio-membrane of diatom cells [36].

Fatty acids are frequently studied among other low-molecular weight metabolites from diatoms. Several metabolites have been suggested to be derived from degradation of fatty acids in which acetyl-CoA acts as intermediate for their biosynthesis [1]. In the present study, significant amounts of essential PUFAs were identified and quantified (*P* ≤ 0.001) to be highly concentrated in the hexane extract compared to the other solvent extracts. The C20, C18, C16 chains including arachidic acid [C20:0] and α-linolenic acid [C18:3] were found to be the prominent fatty acid constituent in the hexane extract, whereas stearic acid [C18:0] and palmitic acid [C16:0] were higher in the acetone and chloroform extracts of *C. calcitrans*. In this regard, the amount of fatty acids produced by the diatom cells may be varied due to differences in growth phase and culture conditions [37]. The abundance of these microalgal lipids were also reported in the earlier study by Sirin et al. [38] in the same species and attracted much interest among microalgal biotechnologists to use as a feedstock for safer biodiesel production. Cholesterol was also detected in the extract of *C. calcitrans*. Generated from squalene biosynthesis, this metabolite is also an important constituent of the biomembrane. Acetone and chloroform extracts had more (*P* ≤ 0.001) cholesterol respectively, compared to the other solvent extracts.

Photosynthetic pigments in diatoms are classified into two classes i.e., carotenoids and chlorophylls. Chlorophylls are derived from geranylgeranyl diphosphate (GPP), whereas geranylgeranyl pyrophosphate (GGPP) led to formation of lycopene and onwards to carotenes and xanthophylls [28]. In this study, the six carotenoids, fucoxanthin, astaxanthin, canthaxanthin, lutein, violaxanthin and zeaxanthin, were significantly more abundant (*P* ≤ 0.001) in the acetone, chloroform and hexane extracts. The carotenoids in diatoms are responsible as health promoters through their capability to prevent inflammation, excessive oxidation and inhibit cancerous cells from proliferating [30,39].

The presence of Chl *a* in diatoms has been reported to act as a mediator for photochemical conversion of solar energy, whereas Chl *c*_1_ in this diatom may serve as accessory pigment in photosynthesis processes [30]. More recently, the finding on Chls-*a* esterified with geranylgeranyl (GG), dihydrogeranylgeranyl (DHGG), and tetrahydrogeranylgeranyl (THGG) found in cells of diatom *C. calcitrans*, was also confirmed by Mizoguchi et al. [40] using ^1^H NMR and ^13^C NMR. The accumulation of these Chls-*a* in *C. calcitrans* might be attributed to high light stress of the organism. Methanol and hexane extracts had significantly (0.010 ≥ *P* > 0.001) higher amounts of Chl *a* and Chl *c*_1_ compared to the other extracts.

## 3. Materials and Methods

### 3.1. Marine Microalgal Material

Dried diatom biomass derived from mass cultures of *C. calcitrans* was provided by the Laboratory of Marine Biotechnology, Institute Biosciences, Universiti Putra Malaysia, and authenticated by Dr. Norio Nagao. The parameters for the microalgal cultivation were also adopted with modifications from Imaizumi et al. [41]. The microalgae was grown with aeration in the presence of light at room temperature in a 10 L conical flask. The microalgal biomass production was cultivated in pure seawater with a salinity of 30 ppt using Conway media that comprised mineral solution, trace metal solution, silicate and vitamin solution (as the basis of nutrients) for the growth of microalgae. The whole growth medium was adjusted to pH 8. After 5–7 days (the stationary phase), this algae was harvested and centrifuged by employing high-speed Sorvall Evolution RC centrifuge (Thermo Electron Corporation, Asheville, NC, USA) at 12,000 rpm at 4 °C, for 5 min. Then, the harvested microalgae was freeze-dried by using ScanVac CoolSafe Freeze Dryer^TM^ (Labogene, Lynge, Denmark) for long-term storage and kept at −80 °C.

### 3.2. Solvents and Chemicals

Analytical grade methanol, ethanol, acetone, chloroform and hexane were purchased from Merck Millipore (Darmstadt, Germany). Acetone-d_6_ and tetramethylsilane (TMS) were also purchased from Merck Millipore (Darmstadt, Germany). For calibration of chemical shifts, 0.1% of TMS was added to the final volume.

### 3.3. Microalgae Extraction Procedure

The lyophilized diatom biomass (100 mg) were dissolved with 50 mL methanol (Me), and vortexed for 5 min. This solution was extracted by sonication for 30 min using ultrasonic waterbath (SK8210HP, Shanghai KUDOS Ultrasonic Instrument Co. Ltd., Shanghai, China) at room temperature. The solvent extract was then filtered through Whatmann No. 1 filter paper while the dried residue was re-extracted with another 50 mL of methanol for a second round of extraction. The extraction process was subsequently repeated in the same manner for a third round of extraction. The filtered extracts were pooled and evaporated to dryness using a rotary evaporator (Heidolph Instruments GmbH amd Co.KG, Schwabach, Germany) at 30 °C, and stored at −20 °C until further analysis. Microalgae extraction was repeated using other solvents i.e., 70% ethanol (70% Et), acetone (Ac), chloroform (Ch) and hexane (He). For each solvent, the extraction was replicated six times.

### 3.4. Sample Preparation for NMR Analysis

The method of Lee et al. [42] with slight modifications was adopted for sample preparation. For measurement of ^1^H NMR spectra of the solvent extracts, approximately 5 mg of extract was dissolved in 600 µL deuterated acetone in a 2 mL Eppendorf tube. For two-dimensional (2D) NMR spectra, 10 mg of extract was dissolved in 600 µL deuterated acetone. In each case, to facilitate solubilization of the extract, the mixture was vortexed for 1 min, ultrasonicated for 15 min, and then centrifuged at 13,000 rpm for 10 min. About 550 µL of the supernatant was then pipetted into a 5 mm NMR tube for the respective NMR measurement.

### 3.5. NMR Analyses

All 1D and 2D NMR spectra were obtained on a 500 MHz Varian INOVA NMR spectrometer (Varian Inc., Palo Alto, CA, USA), running at a frequency of 499.887 MHz at room temperature (25 °C). For each sample, the following parameters were used: set temperature 24 °C, acquisition time for ^1^H NMR spectra 8.49 min, spectral width −2 to 14 ppm, no of scans 28, and relaxation delay 2 s. The presaturation (PRESAT) pulse sequence was applied to all of the samples to reduce water (H_2_O) signals. The 2D NMR experiments i.e., *J*-resolved and ^1^H-^13^C heteronuclear multiple-bond (HMBC) spectroscopies were also carried out to assist biomarker identification.

### 3.6. NMR Spectra Preprocessing and Multivariate Data Analysis

The ^1^H NMR spectra of all samples were automatically binned to ASCII files using Chenomx software (version 6.2, Edmonton, AB, Canada). The spectral region 0.50 to 14.00 ppm was bucketed into 243 integrated regions. The residual signals for water and acetone, in the ranges δ 2.75–3.00 ppm and δ 2.00–2.15 ppm, respectively, were excluded from the analysis. The standardized bucketed data were then pareto-scaled (PAR) and subjected to principal component analysis (PCA) and partial least square analysis (PLS), using SIMCA-P+ software (version 12.0.1.0, Umetrics AB, Umea, Sweden). All 2D NMR spectra were processed using MestRenova (version 6.02-5475, Mestrelab Research, Santiago de Compostella, Spain).

### 3.7. TPC Assay

The total phenolic content assay was carried out according to the methodology described by Lee et al. [41], with slight modifications. Each sample extracts were prepared in dimethyl sulfoxide (DMSO) at a concentration of 100 ppm. Sample extracts or standards of 20 µL were first mixed with 100 µL of Folin-Ciocalteau reagent and allowed the mixture stand for 5 min. Then 80 µL of 7.5% sodium carbonate solution was added. The solutions were incubated in darkness for 2 h and followed by measurement at 765 nm with a Tecan Infinite F200 Pro plate reader (Tecan Group Ltd., Männedorf, Switzerland). The sample extracts were standardized against gallic acid and were expressed as mg GAE/g dw extract.

### 3.8. 2,2-Diphenyl-1-picrylhydrazyl (DPPH) Free Radical Assay

Determination of radical scavenging properties of *C. calcitrans* extracts were performed by following method as described by Lee et al. [42]. 100 µL methanolic DPPH solution was prepared was mixed with 50 µL of sample extracts. Then, the absorbance was taken at 517 nm using a Tecan Infinite F200 Pro plate reader (Tecan Group Ltd., Männedorf, Switzerland) after incubation in the dark for 30 min. Quercetin was used as positive control. All tests were performed in six replicates. The calculation for the scavenging activity was done as *SC*% = [(*A*_o_ − *A*_s_)/*A*_o_] × 100, where *A*_o_ is the absorbance of the reagent blank and *A*_s_ is the absorbance of the sample extracts.

### 3.9. Nitric Oxide (NO) Inhibitory Assay

Inhibition of nitric oxide released by RAW 264.7 cell lines by *C. calcitrans* extracts was quantified using the Griess assay as described by Adebayo et al. [43]. This cultured cell was then activated by incubation in inducer medium containing LPS (10 μg/mL) and 200 units/mL of recombinant murine IFN-_γ_. Cells were incubated under 37 °C in a 5% CO_2_ humidified atmosphere. The cells were split twice prior to seeding in 96 well microtitre plates. Six replicates of microalga of each solvent extraction were subjected to serial dilution in Dulbecco's Modified Eagle's medium (DMEM) media without phenol red containing DMSO (final DMSO concentration of 0.2%). The Griess reagent contains 1% sulfanilamide and 0.1% *N*-(1-naphtyl) ethylene diamine dihydrochloride in 2.5% H_3_PO_4_. Briefly, after 17 h of incubation, 50 µL of the supernatant from the incubated 96-well plates were transferred into new plate and 50 µL of Griess reagent was added. Then, the absorbance was taken at 550 nm using a Tecan Infinite F200 Pro plate reader (Tecan Group Ltd., Männedorf, Switzerland) after incubation in the dark for 15 min.

### 3.10. Cell Viability

To further examine whether the inhibition was not caused by cytotoxic effects, the remaining cells in the incubated wells were measured for cell viability using 3-(4,5-dimethylthiazol-2-yl)-2,5-diphenyltetrazolium bromide (MTT) reagent as previously described by Adebayo et al. [43]. In brief, after media was discarded, 100 µL of DMEM with phenol red was added. Per well, 20 µL of MTT reagent were added and then incubated for 4 h at 37 °C in a 5% CO_2_. Finally, the medium was discarded and 100 µL of DMSO were added. Absorbance was taken at 570 nm after incubation in the dark for 15 min.

### 3.11. Statistical Analysis

The analysis of TPC, DPPH radical scavenging and NO inhibition activities was performed in six replicates. The results were presented as a mean ± standard deviation. Statistical analysis was also performed using Minitab software (Version 17, Minitab Inc, State College, PA, USA) and InStat V2.02 statistical package (GraphPad Prism 7 Software, San Diego, CA, USA). One-way analysis of variance (ANOVA) was used to compare the significant difference between the tested variables. PCA and PLS were performed using SIMCA-P software (v. 13.0, Umetrics, Umeå, Sweden) with Pareto scaling method. Relative quantification was done by manually entering the data into SPSS Data Analysis Version 17.0 (SPSS Inc., Chicago, IL, USA).

## 4. Conclusions

This study has shown that there was a dynamic range of detectable metabolites between acetone, chloroform, methanol, 70% ethanol and hexane extracts from the diatom, *C. calcitrans*. Overall, 29 metabolites were identified from various solvent extracts. Chloroform extract has been shown to be very effective at recovering antioxidant compounds. The results indicated that fucoxanthin, astaxanthin, violaxanthin, zeaxanthin, canthaxanthin and lutein are the metabolites that strongly correlated to both DPPH free radical scavenging and nitric oxide (NO) inhibitory activities. Therefore, NMR spectroscopy combined with MVDA methods could be utilized by various industries to evaluate and obtain maximum-targeted metabolites in a shorter time. In addition to having a high diversity of compounds, the microalgae *C. calcitrans* could serve as important functional food ingredient in the mariculture industry.

## Figures and Tables

**Table 1:** Identified metabolites and its ^1^H NMR (500 MHz, acetone-d*_6_*) assignment of *Chaetoceros calcitrans* in five different solvent extractions.

No	Metabolites	^1^H NMR (Multiplicity)	70% Et *	Me *	Ac *	Ch *	He *
**1**	Glutamic acid	2.39 (m)	+	+	+	+	−
		2.14 (m)	+	+	+	+	−
		2.05 (m)	+	+	+	+	−
**2**	Proline	4.10 (dd)	+	+	+	+	−
		2.35 (m)	+	+	+	+	−
		2.04 (m)	+	+	+	+	−
		1.96 (m)	+	+	+	+	−
**3**	Alanine	1.49 (d)	+	+	+	+	−
**4**	Valine	2.29 (m)	+	+	+	+	−
		1.03 (d)	+	+	+	+	−
		0.98 (d)	+	+	+	+	−
**5**	Isoleucine	0.98 (d)	+	+	+	+	−
		0.94 (t)	+	+	+	+	−
**6**	Leucine	1.69 (m)	+	+	+	+	−
		0.96 (d)	+	+	+	+	−
**7**	Methionine	2.13 (m)	+	+	+	+	−
**8**	Choline	4.05 (ddd)	+	+	−	−	−
**9**	Lysine	1.87 (m)	+	+	+	+	+
		1.73 (m)	+	+	+	+	+
**10**	Glutamine	2.43 (m)	+	+	+	+	+
		2.12 (m)	+	+	+	+	+
**11**	Glycine	3.54 (s)	+	+	+	+	+
**12**	Cholesterol	0.69 (s)	−	−	+	+	−
		0.87 (d)	−	−	+	+	+
		0.88 (d)	−	−	+	+	+
		0.92 (d)	−	−	+	+	+
**13**	Palmitic acid	2.36 (m)	+	+	+	+	+
		1.66 (m)	+	+	+	+	+
		1.29 (m)	+	+	+	+	+
		0.90 (t)	+	+	+	+	+
**14**	Stearic acid	1.77 (t)	−	−	+	+	+
		1.44 (t)	−	−	+	+	+
		1.01 (t)	−	−	+	+	+
**15**	Oleic acid	5.39 (m)	−	−	+	+	+
		2.30 (t)	−	−	+	+	+
		1.96 (m)	−	−	+	+	+
		1.32 (m)	−	−	+	+	+
		0.88 (t)	−	−	+	+	+
**16**	Linoleic acid	5.37 (m)	+	+	+	+	+
		2.36 (t)	+	+	+	+	+
		1.33 (m)	+	+	+	+	-
		0.90 (t)	+	+	+	+	+
**17**	α-Linolenic acid	5.36 (m)	+	+	+	+	+
		2.80 (m)	+	+	+	+	+
		2.35 (t)	+	+	+	+	+
		2.04 (m)	+	+	+	+	+
		1.30 (m)	+	+	+	+	+
		0.96 (t)	+	+	+	+	+
**18**	Arachidic acid	2.35 (t)	−	−	+	+	+
		1.63 (m)	−	−	+	+	+
		1.29 (m)	−	−	+	+	+
		0.88 (t)	−	−	+	+	+
**19**	Glucose	5.20 (d)	+	+	+	+	−
		3.82 (m)	+	+	+	+	−
		3.52 (dd)	+	+	+	+	−
**20**	Sucrose	5.39 (d)	+	+	+	+	−
		4.19 (d)	+	+	+	+	−
		3.82 (m)	+	+	+	+	−
		3.67 (s)	+	+	+	+	−
		3.46 (t)	+	+	+	+	−
**21**	Myo-inositol	4.06 (t)	−	−	+	+	−
**22**	Fucoxanthin	6.81 (dd)	−	−	+	+	−
		6.74 (dd)	−	−	+	+	−
		6.45 (dd)	−	−	+	+	−
		6.43 (d)	−	−	+	+	−
		3.64 (m)	−	−	+	+	−
		2.58 (d)	−	−	+	+	−
		2.31 (dd)	−	−	+	+	−
		2.15 (s)	−	−	+	+	−
		2.07 (s)	−	−	+	+	−
		1.99 (s)	−	−	+	+	−
		1.85 (dd)	−	−	+	+	−
		1.52 (dd)	−	−	+	+	−
		1.38 (dd)	−	−	+	+	−
		1.20 (s)	−	−	+	+	−
		1.08 (s)	−	−	+	+	−
		1.04 (s)	−	−	+	+	−
		0.97 (s)	−	−	+	+	−
**23**	Astaxanthin	6.79 (d)	−	−	+	+	−
		6.20–6.70 (m, olefinic-H)	−	−	+	+	−
		6.51 (d)	−	−	+	+	−
		4.34 (dd)	−	−	+	+	−
		3.67 (s)	−	−	+	+	−
		2.01(s)	−	−	+	+	−
		1.98 (s)	−	−	+	+	−
		1.94 (s)	−	−	+	+	−
		1.82 (t)	−	−	+	+	−
		1.33 (s)	−	−	+	+	−
		1.21 (s)	−	−	+	+	−
**24**	Canthaxanthin	6.20–6.70 (m, olefinic-H)	−	−	−	−	−
		1.86 (s)	−	+	+	+	−
		1.19 (s)	+	+	+	+	−
**25**	Lutein	6.67–6.57 (m, olefinic-H)	−	−	+	+	−
		6.35 (d)	−	−	+	+	−
		6.26 (d)	−	−	+	+	−
		6.07–6.08 (m)	−	−	+	+	−
		5.43 (dd)	−	−	+	+	−
		2.40 (d)	−	−	+	+	−
		1.96 (s)	−	−	+	+	−
		1.91 (s)	−	−	+	+	−
		1.78–1.77 (m)	−	−	+	+	−
		1.74 (s)	−	−	+	+	−
		1.63 (s)	−	−	+	+	−
		1.07 (s)	−	−	+	+	−
		1.01 (s)	−	−	+	+	−
		0.86 (s)	−	−	+	+	−
**26**	Zeaxanthin	1.98 (s)	+	+	+	+	+
		1.97 (s)	+	+	+	+	+
		1.74 (s)	+	+	+	-	+
		1.08 (s)	+	+	+	+	+
**27**	Violaxanthin	1.98 (s)	+	+	+	+	+
		1.94 (s)	+	+	+	+	+
		1.15 (s)	+	+	+	+	−
		0.98 (s)	+	+	+	+	+
**28**	Chlorophyll *a*	9.52 (s)	−	−	+	+	+
**29**	Chlorophyll *c*_1_	9.96 (s)	−	−	+	+	+
		9.90 (s)	−	−	+	+	+
		9.81 (s)	−	−	+	+	−
		8.29(s)	−	−	+	+	−

^*^ Et = ethanol, Me = methanol, Ac = acetone, Ch = chloroform, He = hexane, + = presence, − = absence.

## Supplementary Materials

The following are available online at http://www.mdpi.com/1660-3397/16/5/154/s1, Figure S1: 2D NMR ^1^H (*J*-resolved) spectrum of chloroform extract of *C. calcitrans*, Figure S2: 2D NMR ^1^H-^13^C (HMBC) spectrum of the chloroform extract of *C. calcitrans* (A) at 3.5–10 ppm and (B) at 0.30–3.00 ppm regions, Figure S3: Partial least squares discriminant analysis (PLSDA) (A) score and (B) variable importance in projection (VIP) plots of *C. calcitrans* extracts, Figure S4: PLS derived relationship between Observed Vs Predicted NO (A) and DPPH (B) activity of the chloroform extract of *C. calcitrans*, Table S1: Relative quantification of compounds in the extracts of *Chaetoceros calcitrans*.
