# Supplementary material for: Metabolite Profiling of the Microalgal Diatom Chaetoceros Calcitrans and Correlation with Antioxidant and Nitric Oxide Inhibitory Activities via 1H NMR-Based Metabolomics
Source: Mar Drugs. 2018 May 7;16(5):154. doi: 10.3390/md16050154 (PMC5983285; doi:10.3390/md16050154)
Supplement: Supplementary file 1 [file marinedrugs-16-00154-s001.pdf]

marine drugs

Article

# Supplementary materials: Metabolite profiling of the microalga diatom, *Chaetoceros calcitrans* and correlation with antioxidant and nitric oxide inhibitory activities via $^1$H NMR-based metabolomics

**Awanis Azizan [1], Muhammad Safwan Ahamad Bustamam [1], M. Maulidiani [1], Khozirah Shaari [1,2], Intan Safinar Ismail [1,2], Norio Nagao [3] and Faridah Abas [1,4,*]**

1 Laboratory of Natural Products, Institute of Bioscience, Universiti Putra Malaysia, 43400, Serdang, Selangor Malaysia; Email: awanis_azizan@yahoo.com (A.A.); safwan.upm@gmail.com (M.S.); maulidiani@upm.edu.my (M.M.)

2 Department of Chemistry, Faculty of Science, Universiti Putra Malaysia, 43400 Serdang, Selangor. Email: safinar@upm.edu.my (I.S.I.); khozirah@upm.edu.my (K.S.)

3 Laboratory of Marine Biotechnology, Institute of Bioscience, Universiti Putra Malaysia, 43400 Serdang, Selangor, Malaysia Email: norio_nagao@upm.edu.my (N.N.);

4 Department of Food Science, Faculty of Food Science and Technology, Universiti Putra Malaysia, 43400, Serdang, Selangor, Malaysia; Email: faridah_abas@upm.edu.my (F.A.)

* Correspondence: faridah_abas@upm.edu.my (Faridah Abas)

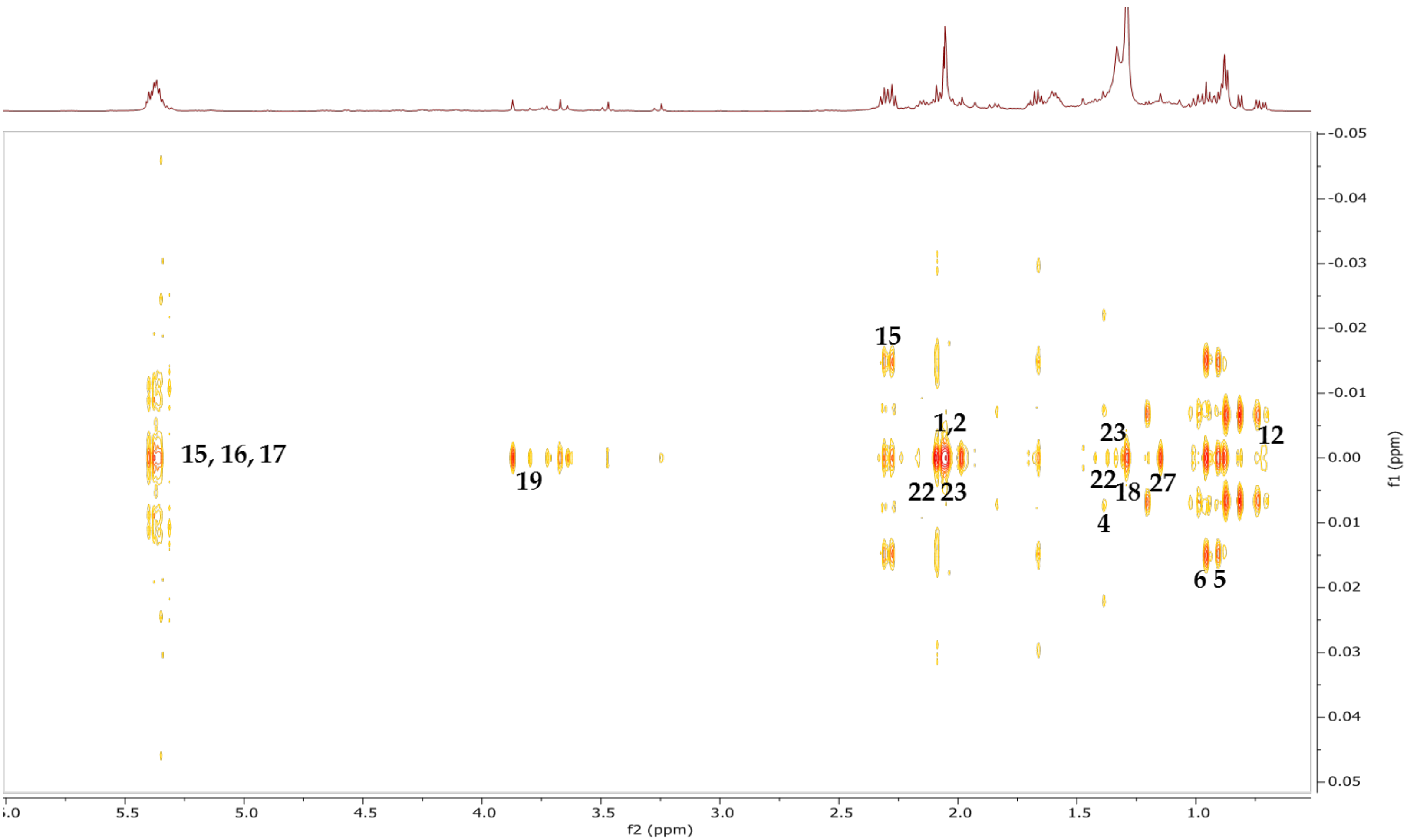


**Figure S1.** 2D NMR $^{1}H$ (*J*-resolved) spectrum of chloroform extract of *C. calcitrans*.

**A**

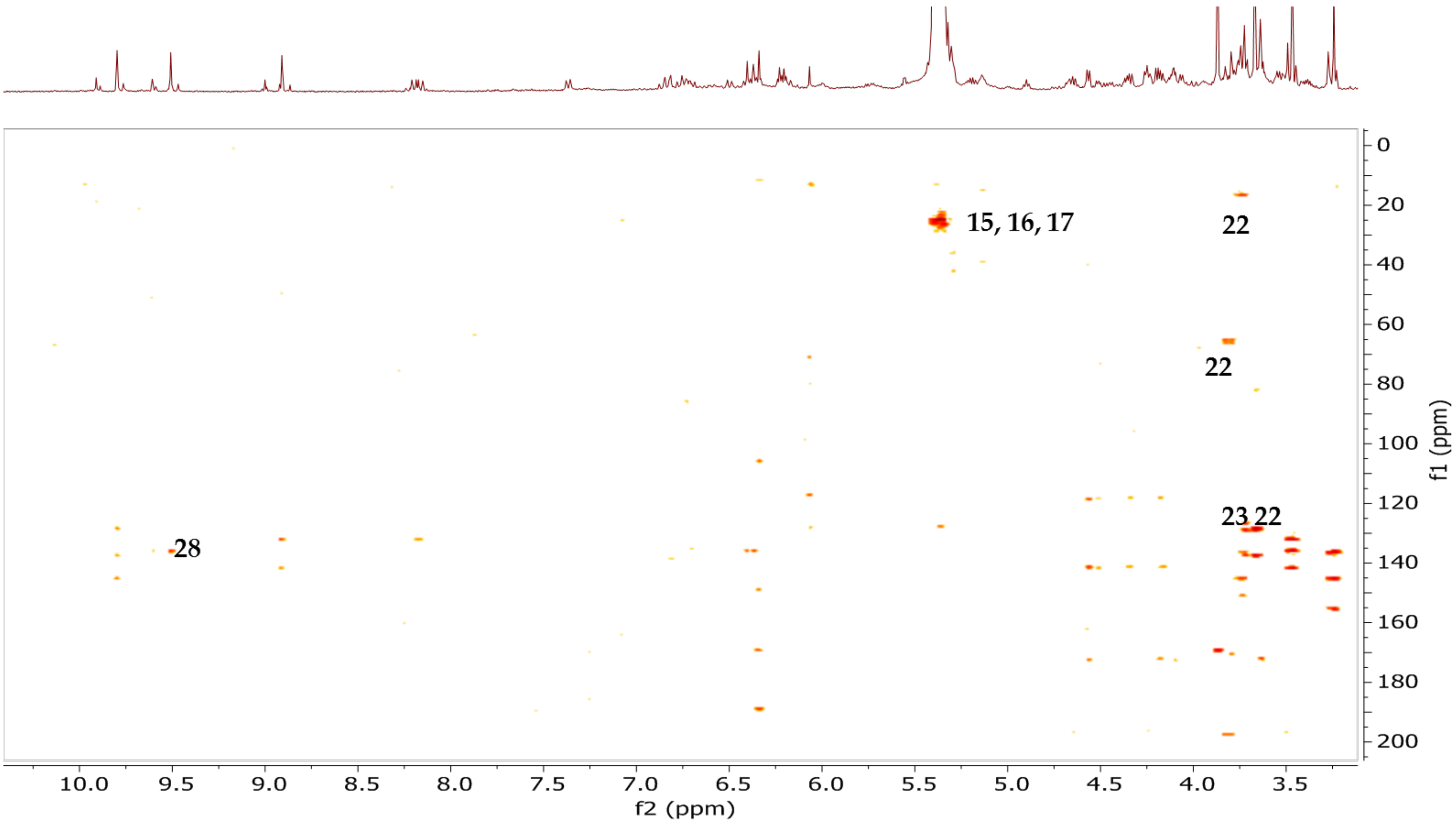


**Figure S2** 2D NMR $^{1}H$-$^{13}C$ (HMBC) spectrum of the chloroform extract of *C. calcitrans* (**A**) at 3.5 – 10 ppm region.

**B**

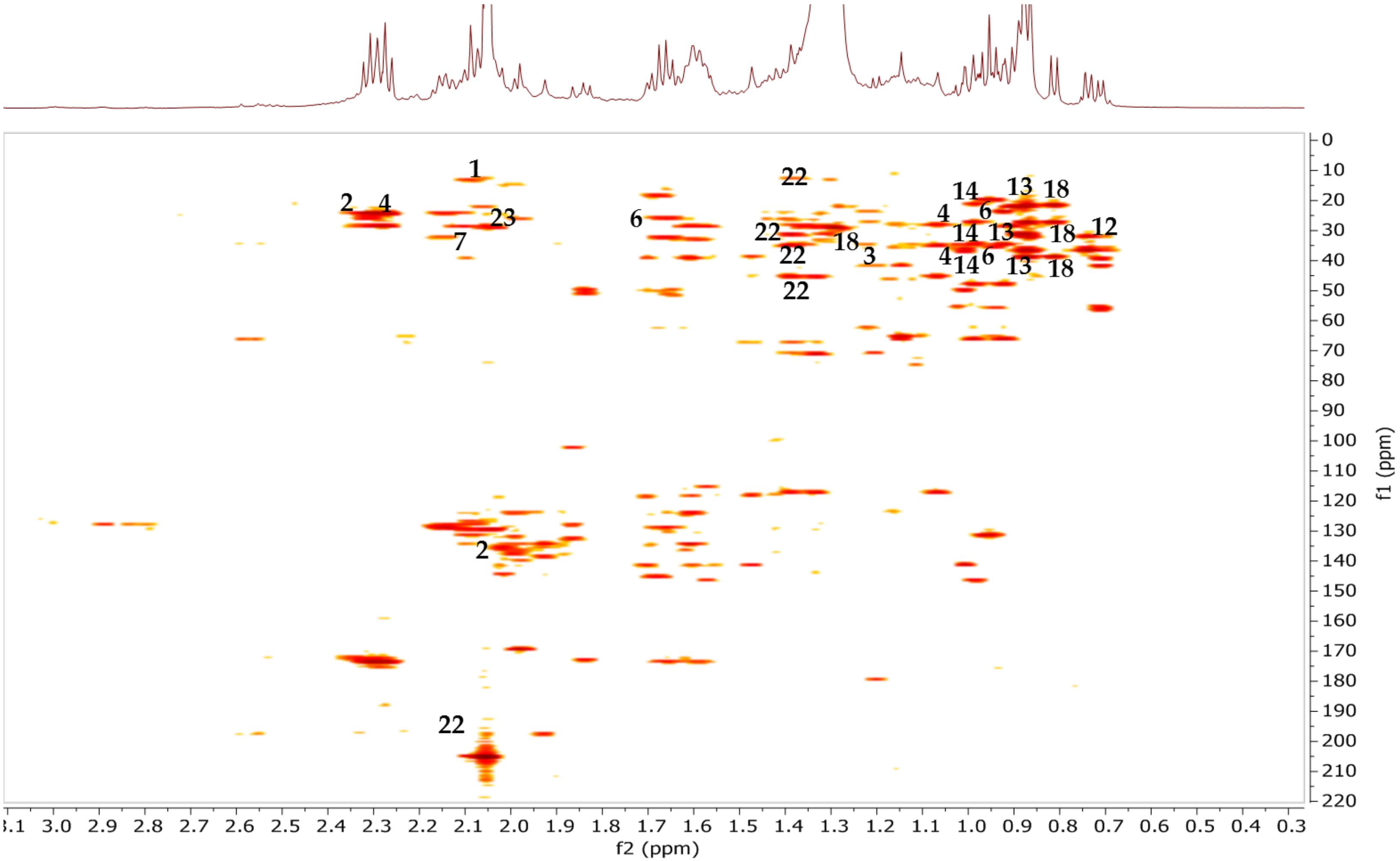


**Figure S2.** 2D NMR $^{1}H$-$^{13}C$ (HMBC) spectrum of the chloroform extract of *C. calcitrans* (**B**) at 0.30- 3.00 ppm region.

**Table S1.** Relative quantification of compounds in the extracts of *Chaetoceros calcitrans*.

| | ***P* value[b]** | | | | | | | | | |
|---|---|---|---|---|---|---|---|---|---|---|
| **Compound** | **A vs C** | **A vs H** | **A vs M** | **A vs 7E** | **C vs H** | **C vs M** | **C vs 7E** | **H vs M** | **H vs 7E** | **M vs 7E** |
| **Chlorophyll-*c1*** | 1.000 | 1.000 | 0.566 | 1.000 | 1.000 | 0.564 | 1.000 | 0.563 | 1.000 | 0.564 |
| **Chlorophyll-*a*** | 1.000 | 0.010 | 0.004 | 1.000 | 0.010 | 0.005 | 1.000 | 0.997 | 0.010 | 0.004 |
| **Arachidic acid** | 0.010 | 0.000 | 0.005 | 0.006 | 0.000 | 0.000 | 0.000 | 0.000 | 0.000 | 1.000 |
| **$\alpha$-Linolenic acid** | 0.022 | 0.000 | 0.011 | 0.012 | 0.000 | 0.000 | 0.000 | 0.000 | 0.000 | 1.000 |
| **Astaxanthin** | 0.000 | 0.000 | 0.000 | 0.000 | 0.000 | 0.000 | 0.000 | 0.000 | 0.000 | 1.000 |
| **Canthaxanthin** | 0.000 | 0.162 | 0.000 | 0.000 | 0.000 | 0.000 | 0.000 | 0.000 | 0.000 | 0.044 |
| **Lutein** | 0.001 | 0.000 | 0.000 | 0.000 | 0.910 | 0.000 | 0.000 | 0.000 | 0.000 | 1.000 |
| **Sucrose** | 0.000 | 0.000 | 0.000 | 0.000 | 0.000 | 0.000 | 0.000 | 0.631 | 0.496 | 0.999 |
| **Palmitic acid** | 0.000 | 0.000 | 0.000 | 0.000 | 0.000 | 0.000 | 0.000 | 0.009 | 0.009 | 1.000 |
| **Fucoxanthin** | 0.676 | 0.009 | 0.005 | 0.006 | 0.156 | 0.000 | 0.000 | 0.000 | 0.000 | 1.000 |
| **Stearic acid** | 0.000 | 0.000 | 0.000 | 0.000 | 0.000 | 0.000 | 0.000 | 0.062 | 0.103 | 0.999 |
| **Isoleucine** | 0.000 | 0.034 | 0.000 | 0.000 | 0.000 | 0.000 | 0.000 | 0.000 | 0.000 | 1.000 |
| **Violaxanthin** | 0.000 | 0.000 | 0.000 | 0.000 | 0.000 | 0.000 | 0.000 | 0.020 | 0.018 | 1.000 |
| **Zeaxanthin** | 0.079 | 0.706 | 0.000 | 0.000 | 0.609 | 0.000 | 0.000 | 0.000 | 0.000 | 0.999 |
| **Cholestrol** | 0.000 | 1.000 | 0.000 | 0.000 | 0.000 | 0.000 | 0.000 | 0.510 | 0.543 | 0.564 |
| **Leucine** | 0.344 | 0.002 | 0.000 | 0.000 | 0.000 | 0.000 | 0.000 | 0.000 | 0.000 | 1.000 |
| **Glucose** | 0.037 | 0.000 | 0.000 | 0.000 | 0.000 | 0.000 | 0.000 | 1.000 | 0.998 | 0.993 |
| **Proline** | 0.000 | 0.000 | 0.000 | 0.000 | 0.000 | 0.000 | 0.000 | 0.988 | 1.000 | 0.984 |
| **Myo-inositol** | 0.000 | 0.000 | 0.000 | 0.000 | 0.000 | 0.000 | 0.000 | 0.754 | 0.634 | 0.102 |
| **Glycine** | 0.425 | 0.000 | 0.000 | 0.000 | 0.000 | 0.000 | 0.000 | 0.999 | 0.926 | 0.983 |

[†] *P* values were results of Tukey-HSD pairwise multiple-comparison tests using SPSS 16.0. Significant level: $P > 0.050$, not significant; $0.050 \geq P > 0.010$, significant*; $0.010 \geq P > 0.001$, very significant**; and $0.001 \geq P$, highly significant***. Letters indicate the three extraction solvents of *Chaetoceros calcitrans*: (A) Acetone, (C) Chloroform, (H) Hexane, (M) Methanol and (7E) 70% Ethanol

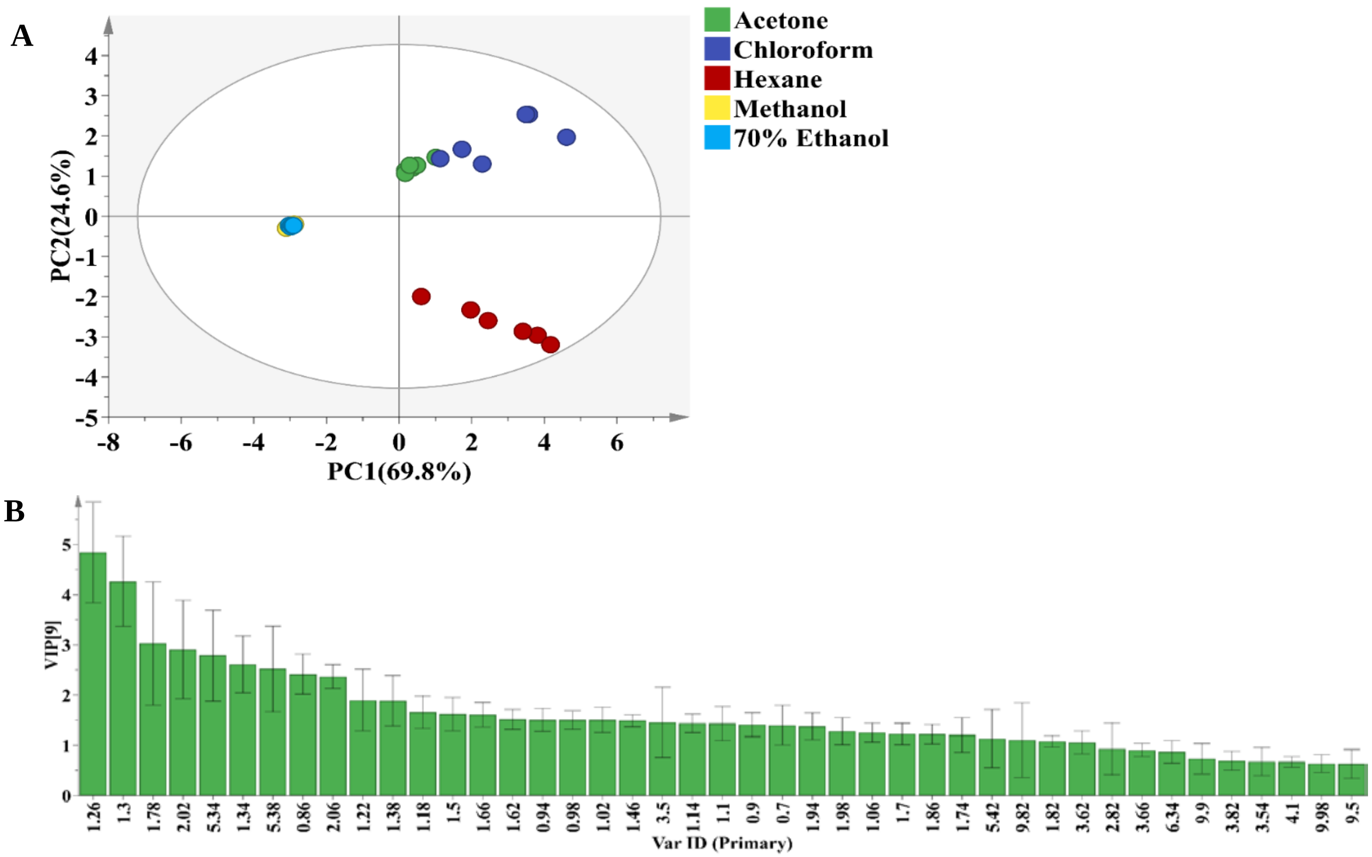


**Figure S3.** Partial least squares discriminant analysis (PLSDA) (**A**) score and (**B**) variable importance in projection (VIP) plots of *C. calcitrans* extracts. Chemical shift (ppm) as listed in VIP plot: arachidic acid (δ 1.26; 1.62), α-linolenic acid (δ 1.3; 2.06; 2.82; 5.34), astaxanthin (δ 1.34; 1.82; 1.94), stearic acid (δ 1.46; 1.78), canthaxanthin (δ 1.18; 1.86 2.02), sucrose (δ 3.66; 5.38), lutein (δ 0.86; 1.02; 1.06; 1.74; 5.42), fucoxanthin (δ 1.22; 1.38; 1.5), palmitic acid (δ 0.9; 1.66), isoleucine (δ 0.94), violaxanthin (δ 0.98; 1.14), zeaxanthin (δ 1.1; 1.98; 6.34), glucose (δ 3.5; 3.82), cholesterol (δ 0.7), leucine (δ 1.7), chlorophyll $c_1$ (δ 9.82; 9.99), myo-inositol (δ 3.62), glycine (δ 3.54), proline (δ 4.1), chlorophyll *a* (δ 9.5).

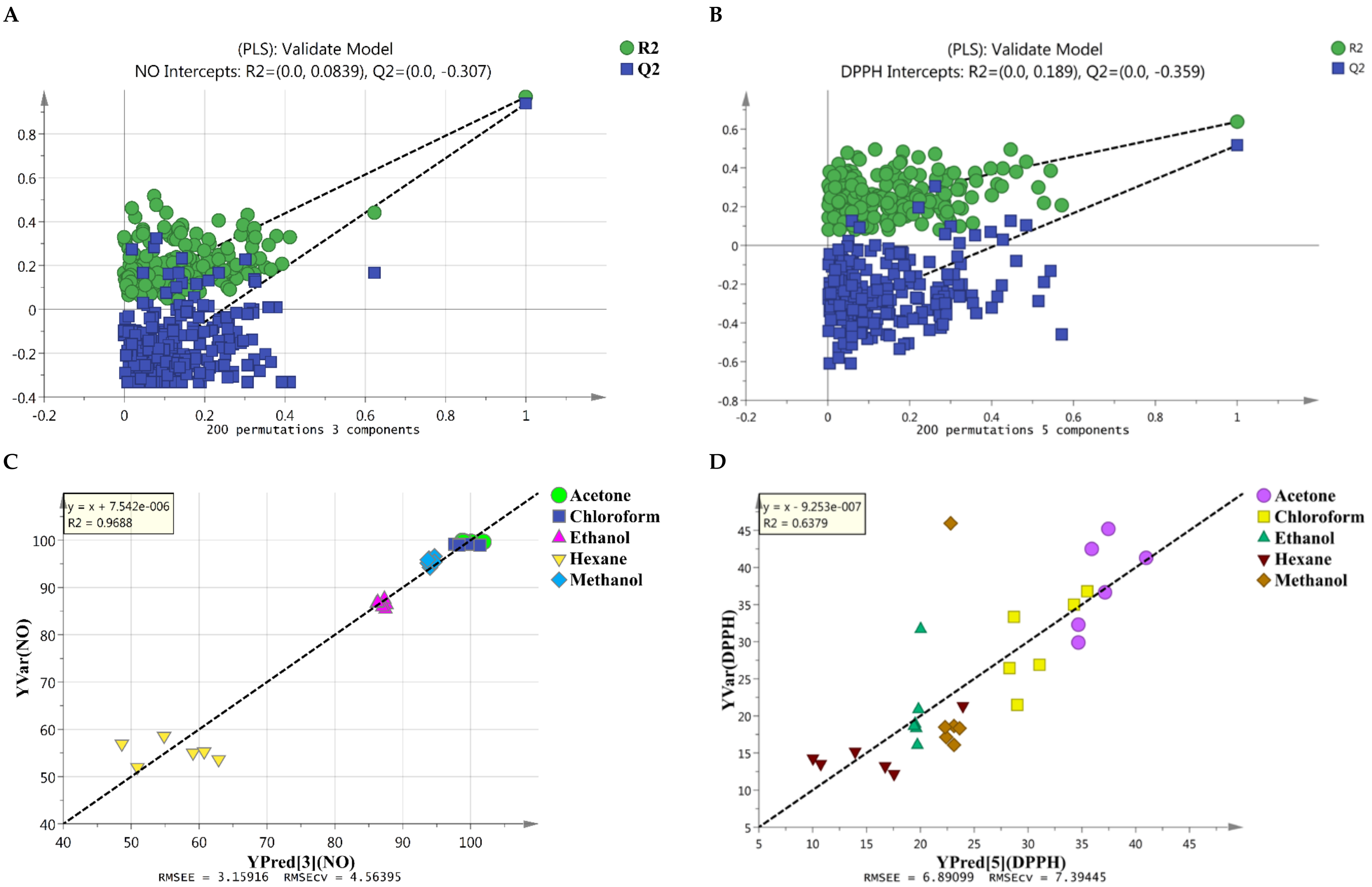


**Figure S4.** Validation of PLS model using permutation test (200 permutations) of NO (**A**) and DPPH (**B**) inhibitory activity. PLS derived relationship between observed vs predicted of NO (**C**) and DPPH (**D**) activity
